# Influence of nickel doping on the moisture adsorption properties of magnesium aluminate spinel: thermodynamic and kinetic analysis

**DOI:** 10.1039/d5ra09380e

**Published:** 2026-02-17

**Authors:** Afsah Saleem, Raouf Hassan, Muhammad Ehtisham, Mansour S. Almatawa, Ahmad K. Badawi, Bushra Ismail

**Affiliations:** a Department of Chemistry, COMSATS University Islamabad Abbottabad Campus-22060 Pakistan bushraismail@cuiatd.edu.pk afsahsaleem110@gmail.com muhammad.ehtisham@udg.edu +92 992 383595 +92 992 383592; b Civil Engineering Department, College of Engineering, Imam Mohammad Ibn Saud Islamic University (IMSIU) 11432 Riyadh Saudi Arabia rahassan@imamu.edu.sa msmatawa@imamu.edu.sa; c Civil Engineering Department, El-Madina Higher Institute for Engineering and Technology Giza 12588 Egypt dr.ahmedkaram91@gmail.com

## Abstract

Magnesium aluminate spinel (MAS) has been widely investigated due to its exceptional properties, which include a high melting point, thermal stability, chemical inertness, abundant vacant sites, substantial porosity, and strong mechanical resilience. The hygroscopic nature of MAS continues to be a major drawback that limits the range of possible uses. In this study, the hygroscopic nature of MAS is investigated for its potential in atmospheric moisture capture. Preliminary thermodynamic and kinetic modeling was performed on experimental data to gain a deeper understanding into the material's surface properties and the fundamental adsorption mechanisms. MAS and Ni doped derivatives were synthesized by a cost-effective coprecipitation method. X-ray diffraction studies confirmed a cubic spinel phase without any impurity phases. Different samples with the general composition of Mg_1−*x*_Ni_*x*_Al_2_O_4_; *x* = 0.2, 0.6, 0.8 were tested on an indigenously designed moisture harvester, and it was found that the adsorption process depends critically on both available humidity and adsorption duration. As the relative humidity varies from 45–85%, Mg_1−*x*_Ni_*x*_Al_2_O_4_ with *x* = 0.2 and *x* = 0.6 exhibited a water adsorption capacity of 1.6 mg g^−1^ to 4.3 mg g^−1^ and 2.7 mg g^−1^ to 12 mg g^−1^, respectively, while at the 45–75% RH, Mg_1−*x*_Ni_*x*_Al_2_O_4_ with *x* = 0.8 exhibited a water adsorption capacity of 8 mg g^−1^ to 13 mg g^−1^. The adsorption capacity was determined by gravimetric analysis using weight measurements of the sample both before and after adsorption. Langmuir, Freundlich, and Temkin adsorption isotherm models were employed to analyze adsorption data. Thermodynamic analysis revealed that adsorption is exothermic and spontaneous, while kinetic studies showed it follows first-order behavior.

## Abstract 2

Atmospheric water capture on Ni^2+^-doped derivatives of magnesium aluminate spinel in an indigenously-built reactor.

## Graphical abstract

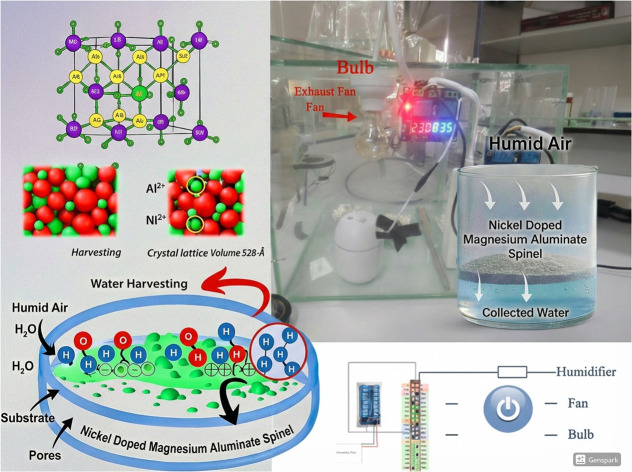

## 1. Introduction

Water scarcity is a pressing issue affecting people worldwide. As a result, many individuals are forced to rely on contaminated water sources. Unfortunately, this polluted water can lead to waterborne diseases, which can be fatal.^1,2^ The increasing droughts and floods caused by global warming are making the situation even worse, disrupting our traditional freshwater sources. This really emphasizes how crucial it is to develop climate-resilient and alternative water delivery technologies.^3,4^ Seawater desalination and the treatment of polluted water sources are two alternate water supply options that have been investigated.^5,6^ However, these techniques can be quite costly, and the disposal of brine from desalination poses a significant challenge.^7,8^ The enormous reservoir of over 13 000 km^3^ of water vapor in the atmosphere (six times the quantity of freshwater available on Earth), has made atmospheric water harvesting (AWH) more popular in recent years.^9–11^

**Fig. 1:**
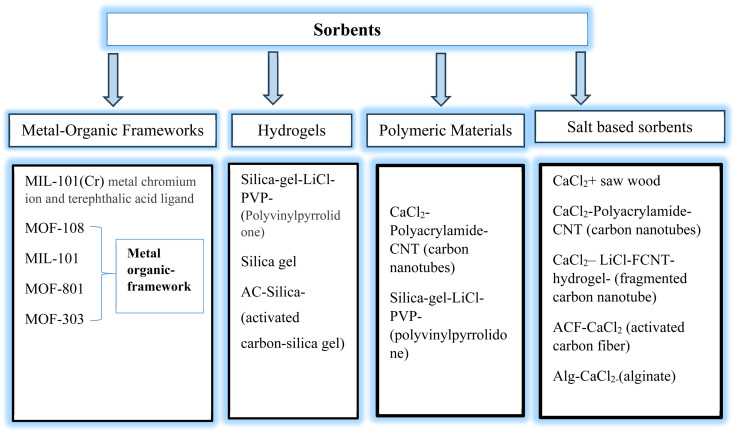
Common adsorbents used in atmospheric water harvesting.^5,10,13,14^

There are a variety of technological methods, each utilizing a unique way to capture and condense moisture from the atmosphere, that can be employed for atmospheric water harvesting (AWH).^9,11^ Some of these include fog harvesting, dew water harvesting, the vapor compression cycle, and sorbent-based atmospheric water harvesting (SBAWH).^8^ Each of these techniques has its own set of difficulties; for example, the vapor compression cycle often uses a lot of energy, while fog and dew harvesting are significantly impacted by geographic location.^11,12^ Conversely, SBAWH has a high adsorption capacity and is reasonably priced and environmentally beneficial.^8,11^ However, a number of variables affect its efficacy, including the adsorbent's surface affinity, the size per volume of its pores, and the surrounding temperature and relative humidity (RH). A thorough literature survey is conducted for the various adsorbents used in AWH such as zeolite, silica gel, metal–organic frameworks (MOFs), covalent organic frameworks (COFs), hydrogels, and composite materials. We classified these materials into four broader categories as shown in Fig. 1. These adsorbents present inherent limitations and challenges that must be addressed. Although MOFs have a large surface area and adjustable porosity, they can be expensive, have poor hydrolytic stability, and require complicated preparation.^1,11^

On the other hand, polymers often exhibit slow adsorption rates, while hygroscopic salts can experience deliquescence and poor long-term stability. These drawbacks underscore the continuous need for inexpensive, structurally sound sorbents that combine superior adsorption kinetics, long-term durability, and high water adsorption capacity.

We have compiled the water uptake capacities of several materials in Table 1 below to better highlight the need for ongoing research. Table 1 shows that the water uptake capacities of different sorbent materials described in the literature vary significantly based on the sorbent type, structure, and environmental circumstances. While MOFs and salt-based composites are known for their impressive water absorption capabilities, most of these technologies are still stuck in the lab or pilot phases. Only a handful are ready for the market, mainly due to challenges like cost, stability, and scalability. On the other hand, the application of silica-based sorbents and other stable oxide materials in small-scale solar-powered water harvesting systems has been limited, highlighting the urgent need for affordable, durable, and high-capacity alternatives.

**Table 1:** Water uptake performance of various adsorbent materials reported in the literature

Material	Water uptake	Reference
CaCl_2_ + saw wood	500 ml m^−2^ day^−1^, 2.8 kg (0.025 ml^−1^ 0.1 g day^−1^)	15
MIL-101(Cr)	15.9 L kg^−1^ day^−1^ (1.59 ml^−1^ 0.1 g day^−1^)	14
MOF-108	2.8 L kg^−1^ at RH of 20% (0.28 ml 0.1 g^−1^)	5
MIL-101(Cr)	15.9 L kg^−1^ day^−1^ (1.59 ml^−1^ 0.1 g day^−1^)	14
MOF-108	2.8 L kg^−1^ at RH of 20% (0.28 ml 0.1 g^−1^)	5
Silica-gel-LiCl-PVP	0.43 g g^−1^ (0.043 g 0.1 g^−1^)	10
Silica gel	1.5 to 3.3 L day^−1^ per square meter of solar field	
AC-silica (activated carbon-silica gel)	0.81 kg kg^−1^ (0.081 g 0.1 g^−1^)	16

As a result, recent research is increasingly focused on identifying sorbents that not only demonstrate strong adsorption capabilities but are also economically viable and environmentally friendly.^1^ In this sense, both industry and academia have recently paid close attention to magnesium aluminate spinel.^17^ This is largely due to its impressive combination of properties, including high mechanical strength at room temperature (ranging from 135 to 216 MPa), remarkable hardness (16 GPa), and a relatively low density of 3.58 g cm^3^.^10,17^ This material can handle high temperatures quite well, showing impressive strengths ranging from 120 to 205 MPa at 1300 °C, and it boasts a melting point of 2135 °C. The most fascinating is its unique crystal structure, which features a cubic close-packed (face-centered cubic, FCC) arrangement of oxide anions (O^2−^), making it a potential candidate for water harvesting. In this FCC lattice, the cations Mg^2+^ and Al^3+^ fill specific interstitial positions: Mg^2+^ typically occupies one-eighth of the tetrahedral sites, while Al^3+^ takes up half of the octahedral sites. The distinctive stoichiometry arises from the arrangement of 8 AB_2_O_4_ formula units per unit cell, providing a robust framework for doping and enhancing functional characteristics.^8,18^ The unique feature of the spinel structure is that each unit cell has 72 vacant sites that can hold a sizable number of divalent and trivalent cations in a solid solution without reducing the crystallographic geometry.^6^

The hygroscopic nature of this material is often viewed as a limitation in traditional uses, even though there's a wealth of research on the topic. Instead, studies have focused on enhancing synthesis methods, boosting mechanical strength, and more. The unique lattice structure of spinel, with its numerous unoccupied tetrahedral and octahedral sites, allows for the accommodation of dopant cations, which can lead to customizable characteristics. With its high structural stability, the ability to adjust structural and morphological properties through doping (like with Ni), and its natural hygroscopic properties, MAS stands out as a promising choice for durable and effective water adsorbents. This is especially important as we look for scalable and cost-effective materials for AWH systems. It's worth noting that, despite its many advantages, MAS hasn't been explored for water collection applications until now, which really underscores the originality and potential impact of this study.^8,10^

This study's main goal was to synthesize nanoscale Ni-doped magnesium aluminate spinel derivatives having the general formula Mg_1−*x*_Ni_*x*_Al_2_O_4_ (*x* = 0.2, 0.6, 0.8) using a simple co-precipitation technique in order to take advantage of its designed defect structure and greatly increase water adsorption capacity. A significant innovation of this work that provides insights beyond conventional adsorbents is the correlation between enhanced water adsorption efficiency and the structural and morphological characteristics of Ni-doped MgAl_2_O_4_. The synthesized material was carefully evaluated for its adsorption behavior under controlled settings in order to comprehend water-adsorbent interactions with spinels. Thermodynamic analysis supported the interpretation of the data using Langmuir, Freundlich, and Temkin isotherms.

## 2. Experimental studies

### 2.1 Synthesis

For the synthesis of spinels, the co-precipitation approach has long been used. In addition to being reasonably priced, it produces excellent results and is quite practical, saving you a great deal of time. This procedure entails adding an alkaline NaOH solution in accordance with eqn (1) and combining a stoichiometric amount of the necessary metal salt solution with constant vigorous stirring. When creating different spinel compositions, the pH level is critical, so it is critical to monitor it closely to make sure you have pure spinel material.^17^ Following chemicals were used as recieved: Mg(NO_3_)_2_·6H_2_O (98.97%, Daejung), Al(NO_3_)_2_·9H_2_O (98%, Daejung), Ni(NO_3_)_2_·6H_2_O (98.99%, Daejung), and NH_3_·H_2_O (Honeywell 33% NH_3_). To start, 100 ml of a 0.1 M aqueous solution of Mg(NO_3_)_2_·6H_2_O with 0.2 M Al(NO_3_)_2_·9H_2_O were mixed together. The precipitation process involved adding 2 M NH_3_·H_2_O while keeping the pH at 9. After an hour of stirring to produce precipitates at room temperature, they were cleaned and dried in an oven for an additional hour before being annealed for eight hours at 800 °C in a muffle furnace. With a heating rate of 5 °C min^−1^ to obtain the crystalline spinel phase. For the Ni^2+^ doped derivatives, which follow the general formula Mg_1−*x*_Ni_*x*_Al_2_O_4_ (where *x* = 0.2, 0.6, 0.8), stoichiometric amounts of Ni(NO_3_)_2_·6H_2_O salt solutions were added, adhering to the same procedure.1
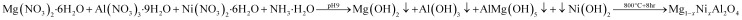


For doped derivatives, the obtained precipitates were stirred at room temperature for one hour, washed, oven-dried for an additional hour, and subsequently annealed in a muffle furnace at 800 °C for 8 h with a heating rate of 5 °C min^−1^ to obtain the crystalline spinel phase further details about the synthesized compositions can be found in supplementary data as shown in Fig. S1. After synthesis, the powders were evaluated for water adsorption performance. All adsorption experiments were conducted in triplicate (*n* = 3) to ensure reproducibility. The obtained data were statistically analyzed and are presented as mean ± standard deviation.

### 2.2 Characterization

A Shimadzu XRD-7000 diffractometer operating at 40 kV with Cu Kα radiation (*λ* = 1.5406 Å) was used to record X-ray diffraction (XRD) patterns. To analyze the surface crystallinity and phase composition of the synthesized material, scans were conducted over a 2*θ* range of 15–70° with a step size of 0.02. Structural parameters such as the lattice constant ‘*a*’ (Å), unit cell volume ‘*V*_cell_’ (Å^3^), Scherrer crystallite size ‘*D*_s_’ (nm), and X-ray density ‘*ρ*_X-ray_’ (g cm^−3^) were evaluated using the equations below^19^ with “*d*” spacing values, and “*h*”, “*k*”, “*l*” are the miller indices.

2 *a* = [*d*^2^ (*h*^2^ + *k*^2^ + *l*^2^)]^1/2^

3 *V*_cell_ = *a*^3^

Scherer's equation is given by formula4
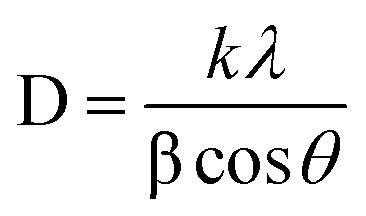
where “*λ*” is the wavelength of X-rays, “*β*” is FWHM (full width at half maximum) of each peak, value of shape factor “*k*” is 0.94 “*θ*” represents the Bragg's angle.

X-ray density (*ρ*_X-ray_) is given by formula.5
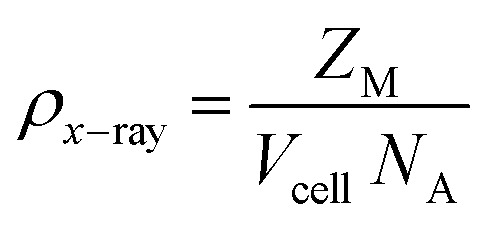


SEM images of the synthesized Mg_1−*x*_Ni_*x*_Al_2_O_4_ samples were recorded using a JEOL JSM-5910 scanning electron microscope (Japan). Scanning electron microscopy gives detailed information about material's morphology and composition. In the scanning electron microscope, a high-energy beam of electrons interacts with the material surface, this type of interaction shows refraction, reflection, and scattering to produce the data in the form of signals.^20^ The energy dispersive X-ray spectroscopy (EDX) coupled with SEM is mainly used for elemental analysis. It is basically interaction of source of X-rays with the sample resulting in the excitation. The capabilities of excitation is as a result of fundamental principle that it show unique pattern of atomic structure by emission of characteristic X-rays.^21^

### 2.3 Adsorption studies

**Fig. 2:**
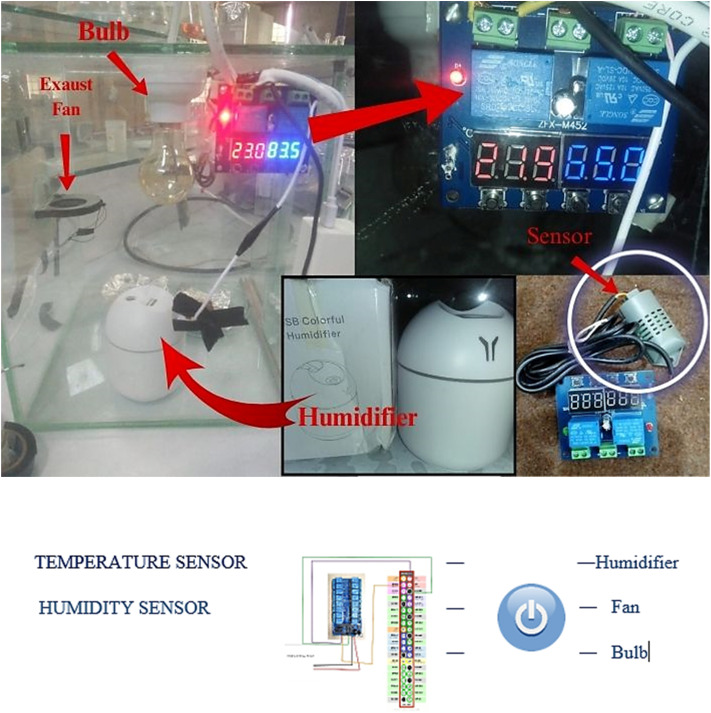
Atmospheric water harvesting equipment.

A closed rectangular glass container that is 12 cm tall and 10 cm wide is part of the experimental setup (Fig. 2). Because of its airtight design, the internal environment is kept constant and unaffected by outside changes. There is a colorful, USB-connected humidifier inside that lets us set the humidity levels we want by just pressing a button. The relative humidity that this humidifier can provide ranges from 45% to 95%. A relay system is also in place to immediately monitor the chamber's temperature and humidity levels. We are able to perform the adsorption procedure efficiently because of this controlled atmosphere. A fan at the rear of the chamber helps to remove any excess moisture in order to control excessive humidity. The procedure for the adsorption studies was as follows.^11^ First, the sample was placed in an oven to eliminate moisture content at a temperature of 200 °C for 2–3 hours. This step was repeated before each experiment. Once dried, the sample was weighed on an analytical balance to establish its initial weight. For the adsorption calculations, we needed at least 0.1 g of the sample, which was weighed on aluminum foil. After recording the initial weight, the sample was placed inside the chamber, where humidity was maintained at 45% using the humidifier and fan. After 20 minutes, we monitored the sample's weight again with the analytical balance. This process was repeated multiple times (from 20 to 120 minutes) to gain a deeper understanding of the adsorption mechanism. The moisture content was determined by calculating the difference between the initial and final weights of the sample. We followed the same procedure for all samples at humidity levels ranging from 55% to 95% relative humidity.

### 2.4 Isotherm modeling

When the temperature remains constant, the balance achieved through the adsorption of substances on a material's surface is primarily characterized by adsorption isotherms. The substances that need to be removed during the adsorption process are referred to as adsorbates, while the material that facilitates this process is known as the adsorbent. Once equilibrium is reached under constant temperature conditions, the adsorption isotherms serve as mathematical expressions that illustrate the relationship between the amount of adsorbate being adsorbed on the adsorbent's surface and the concentration of the adsorbate in the solution.^15^ The concept of a monolayer, which indicates the maximum adsorption capacity, is explained by the Langmuir adsorption isotherm. This isotherm is based on several key assumptions: the adsorption of adsorbate occurs at fixed reaction sites on the adsorbent, with each site capable of holding only one adsorbate at a time. Additionally, all reaction sites are assumed to have the same energy, and there is no interaction between one adsorbate and another, nor with the adsorption sites.

Langmuir adsorption isotherm is indicated by the following eqn (6).6
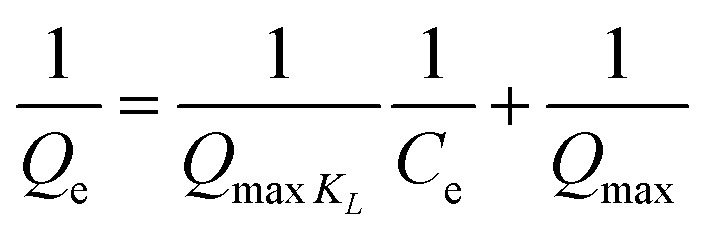
where “*Q*_e_” is the amount of adsorbed molecule of adsorbate per gram of adsorbent (mg g^−1^), “*Q*_max_” is the monolayer adsorbent capacity (mg g^−1^), “*C*_e_” is the equilibrium concentration of adsorbate (mg g^−1^), and “*K*_L_” is constant of Langmuir adsorption.

Freundlich adsorption isotherm assumes that surface is heterogeneous in nature. It defines the physisorption that is strong in nature and forms multilayers. Freundlich adsorption isotherm is represented by the eqn (7).7
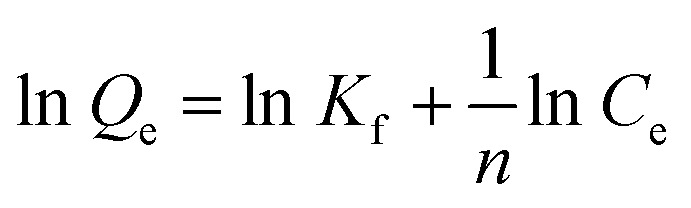
where “*K*_f_” is constant of Freundlich adsorption isotherm, “*C*_e_” is the equilibrium point concentration of adsorbate (mg g^−1^), “*Q*_e_” is the concentration of adsorbate that is adsorb on adsorbent (mg g^−1^), and *n* describes the linearity of adsorption process between adsorbate and adsorbent.

Temkin adsorption isotherm is represented by the eqn (8).

8 *Q*e = *B*_T_*L*_*n*_*A*_T_ + *B*_T_ ln *C*_e_

where *B*_T_ is the heat constant of adsorption (if the *B*_T_ < 8 kJ mol^−1^, it indicates the physical adsorption *A*_T_ is the equilibrium binding constant of adsorption, and *T* is the absolute temperature.^22^

### 2.5 Kinetic studies

The influence of time on the process of adsorption is mainly due to the kinetic activity of adsorbate on the adsorbent surface.^12^

Pseudo first order kinetics informs about the physical adsorption. It demonstrates about the weak forces of attraction between adsorbate and adsorbent. The Lagergren first order kinetics based upon the amount of adsorbate that are being adsorbed, while the difference in the process of adsorption is mainly due to the driving force of the adsorption process. The first-order kinetic model is described as under.^12^

9 *M*_*t*_ = *M*e + (*M*_0_ – *M*e)e^−*k*_1_*t*^

whereas the rate constant “*k*_1_” is the adsorption rate constant for the first-order kinetic model (h^−1^).

A second-order kinetic model is based on the fact that there is an involvement of chemical bonding among adsorbate and adsorbent. EMC “*M*_e_” can be calculated by this model as.^12^10
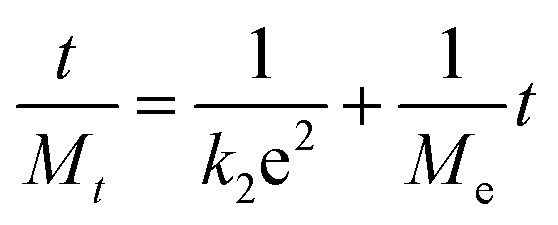
whereas the rate constant “*k*_2_” is the rate constant for the second order kinetic model (h^−1^).

### 2.6 Thermodynamic studies

The feasibility of the reaction was investigated by using thermodynamic parameters.^12^

11 *Q*_st_ = *q*_st_ + *H*_L_

The net isosteric heat of sorption is needed to change the state of liquid to vapors, with the water activity(*a*_w_). Experimentally, the isosteric heat of adsorption can be obtained from the Clausius–Clapeyron relationship by constructing a plot of ln(water activity) *versus* 1/*T*.12
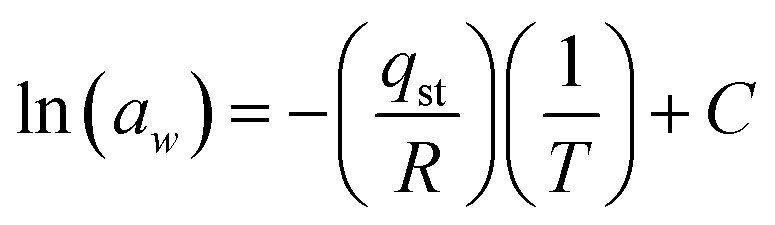
Here, *T* is the absolute temperature (K), *R* is the gas constant (8.314 J mol^−1^ K) “*q*_st_” is the net isosteric heat of sorption (J mol^−1^) and *C* is the constant.

The change in entropy plays a crucial role in the system's energy analysis, being proportional to the number of sorption sites available at a particular energy level. The differential sorption entropy (Δ*S*) was obtained by fitting eqn (13) to the equilibrium moisture content derived from the fitting.^12^13
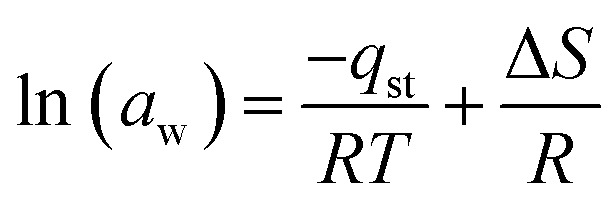

14 Δ*G* = *RT* ln *a*_w_

## 3 Results and discussion

### 3.1 Characterization

**Fig. 3:**
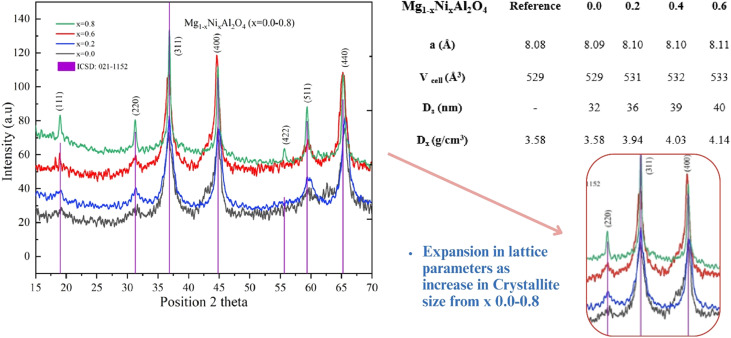
X-ray diffraction pattern of Mg_1−*x*_Ni_*x*_Al_2_O_4_ (*x* = 0.0, 0.2, 0.6, 0.8).

The observed diffraction pattern and corresponding Miller indices (*hkl* values) align closely with the standard pattern for Mg_1−*x*_Ni_*x*_Al_2_O_4_ (ICSD code no. 021-1152, Fig. 3), with prominent peaks identified at (111), (220), (311), (400), (422), (511), and (440), confirming a single-phase composition with *Fd*3*m* space group symmetry. These peak values confirm that the Mg_1−*x*_Ni_*x*_Al_2_O_4_ (*x* = 0.2, 0.6, 0.8) has a single-phase composition with *Fd*3*m* space groups. In particular, earlier works on Mg_1−*x*_Ni_*x*_Al_2_O_4_ have also shown that increasing Ni^2+^ content does not disrupt the spinel framework and that diffraction intensities evolve systematically with doping level, confirming successful Ni substitution into the MgAl_2_O_4_ lattice.^23^ Through X-ray diffraction analysis, several parameters were determined, including cell volume (*V*_cell_), X-ray density (*D*_X_), and crystallite size (*D*_s_). For Mg_1−*x*_Ni_*x*_Al_2_O_4_ (*x* = 0.2), the calculated values are a cell volume of 531 Å^3^, bulk density of 3.94 g cm^−3^, and a crystallite size of 36 nm. For Mg_1−*x*_Ni_*x*_Al_2_O_4_ (*x* = 0.6), the values are 532 Å^3^ for cell volume, 4.03 g cm^−3^ for bulk density, and 39 nm for crystallite size. Lastly, for Mg_1−*x*_Ni_*x*_Al_2_O_4_ (*x* = 0.8), the values are 533 Å^3^ for cell volume, 4.14 g cm^−3^ for bulk density, and 40 nm for crystallite size. This trend of increasing lattice parameters is attributed to the larger ionic size associated with higher nickel content in magnesium aluminate spinel. It suggests that higher nickel content has a strong preference for occupying the tetrahedral sites, which correlates with the increase in crystalline size. The magnesium aluminate (Mg_1−*x*_Ni_*x*_Al_2_O_4_) is doped with divalent nickel (*x* = 0.2, 0.6, and 0.8) at these tetrahedral sites. Notably, no additional peaks were observed in any of the samples apart from those corresponding to the spinel phase, confirming that the doped spinel compounds indeed generate a single-phase spinel structure.

**Fig. 4:**
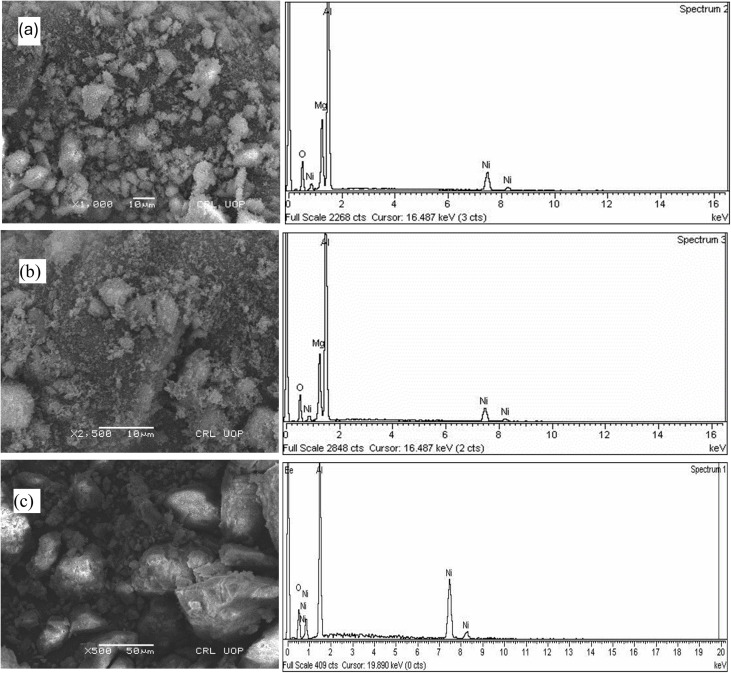
SEM micrographs and EDS spectra of (a) Mg_1−*x*_Ni_*x*_Al_2_O_4_ (*x* = 0.2), (b) Mg_1−*x*_Ni_*x*_Al_2_O_4_ (*x* = 0.6), (c) Mg_1−*x*_Ni_*x*_Al_2_O_4_ (*x* = 0.8).

This study shows how the host lattice magnesium aluminate (MgAl_2_O_4_) can take in impurity ions without altering its spinel structure (Fig. 4). We see that the broadening of the peak improves, indicating that the doped materials form tiny crystallites. As we increase the amount of dopant, both the lattice constant (*a*) and the cell volume gradually rise. Similarly, the grain size of the doped samples also increases steadily with more content. Additionally, the X-ray density of the doped compounds goes up as the concentration of the dopant increases. This is because the dopant cations have a larger molar mass, leading to a consistent increase in the density of the doped samples as the content rises.

The EDS spectrum for Mg_1−*x*_Ni_*x*_Al_2_O_4_ (where *x* = 0.2, 0.6, 0.8) is presented, showing that the theoretical composition aligns well with the experimental findings. Scanning electron micrographs revealed relatively uniform particle distribution with typical spinel morphology. Similar observations have been reported for Ni-doped magnesium aluminate spinels, where SEM revealed homogeneous particles and surface textures that can influence adsorption behavior.^24^ The SEM image of Mg_1−*x*_Ni_*x*_Al_2_O_4_ (*x* = 0.2, 0.6, 0.8) was taken after it was annealed for eight hours at 800 degrees Celsius. The morphological features indicate uneven growth, with varying particle sizes, and some regions exhibit pores while others do not. The micrographs also highlight a rough surface texture with significant porosity, suggesting that the material's tendency to absorb moisture led to the aggregation of particles. Interestingly, the SEM images do not show any noticeable difference in particle size due to the tetrahedral doping of Ni^2+^ cations; however, the slightly rough appearance of the samples indicates that larger particles have formed on the surface as a result of smaller crystallites coming together. Within the limits of experimental error, the EDS spectrum shows a strong correlation between the theoretical and experimental compositions.

### 3.2 Adsorption studies

**Fig. 5:**
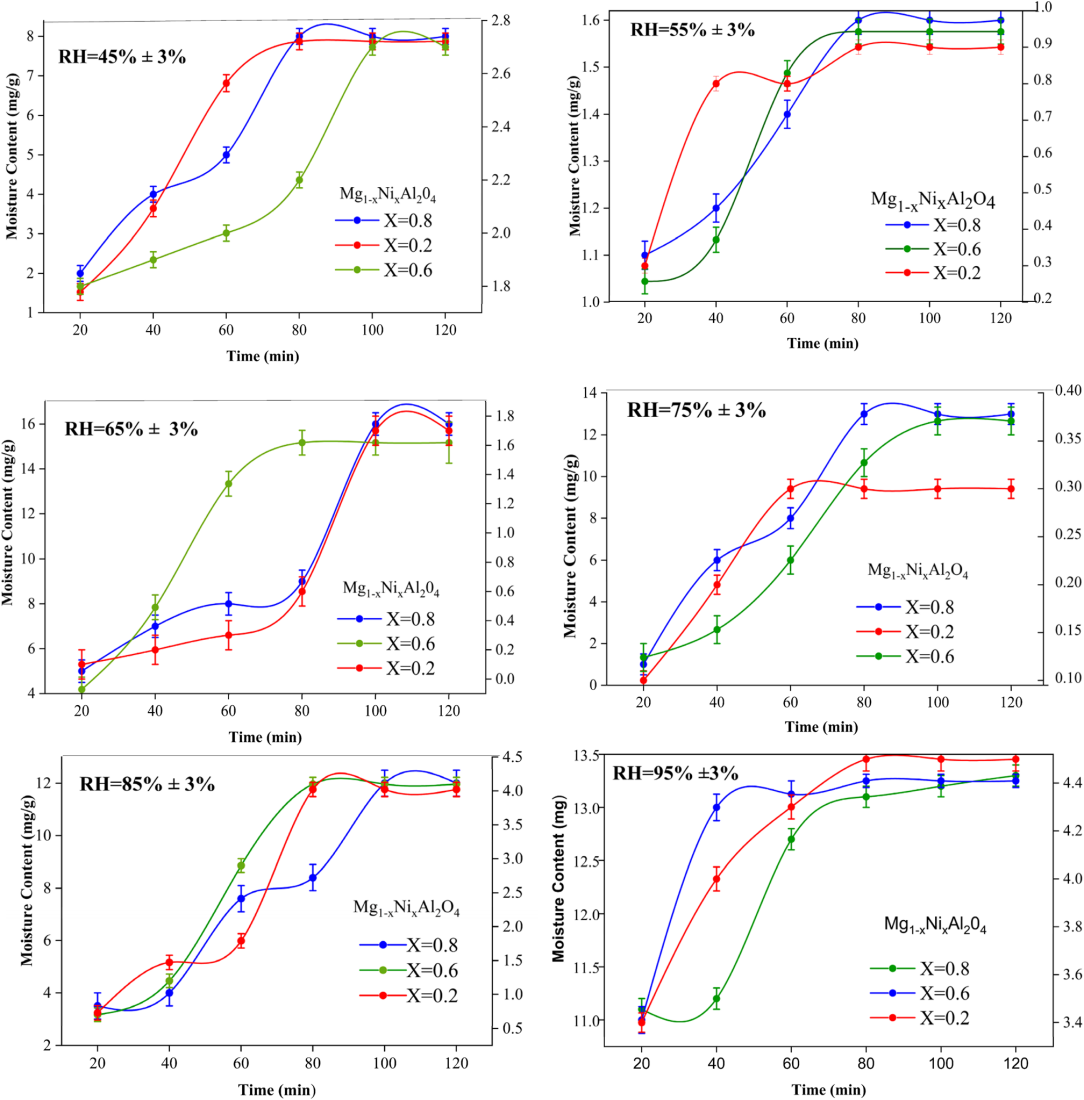
Moisture content *versus* time for synthesized Mg_1−*x*_Ni_*x*_Al_2_O_4_ (*x* = 0.2, 0.6, 0.8) at different relative humidity (RH%).

The capability of Mg_1−*x*_Ni_*x*_Al_2_O_4_ (*x* = 0.0, 0.2, 0.6, 0.8) to adsorb moisture content was measured by plotting a graph between moisture content *versus* time with the help of origin software and Microsoft EXCEL. Initially, there is a steep trend observed in all the graphs because a large number of vacant sites are available as shown in Fig. 5.

The equilibrium moisture content is the phenomenon at which no adsorption–desorption takes place. It is the state where the adsorbent has reached equilibrium between uptake and release of molecules. At different relative humidity, the trend of equilibrium moisture content changed depending upon the increased number of adsorbed vapors at higher humidity. At lower moisture, the pressure caused by the vapors on the adsorbent surface is lower as compared to higher humidity so that's why EMC and saturation point achieves earlier. After saturation point, the moisture content starts decreasing this may be due to the reason that the vapor pressure on the material surface is higher as compared to the surrounding area. So may be some of vapors get desorbed from the material surface.

The equilibrium moisture content of Mg_1−*x*_ Ni_*x*_Al_2_O_4_ with different compositions on various water activity values. It can be seen from Fig. 5 that *x* = 0.8 shows high values of moisture content this may be due to the increased number of defects. It shows that at *x* = 0.8, equilibrium moisture content was achieved at low water activity values very easily compared with *x* = 0.0 that reached EMC at very high-water activity. This is due to the reason that the diffusion rate of water molecules to undergo physical adsorption is high, but on the other hand at high water activity values, the equilibrium moisture content value decreases due to decreased rate of diffusion.

In case when *x* = 0.6, with increase in water activity, the equilibrium moisture content decreases, then gain stability and increases with the increase of water activity and become maximum at the higher water activity value. Thus, composition *x* = 0.6 provides a greater number of active sites for adsorption, thus equilibrium moisture content obtains at the higher value of water activity.15
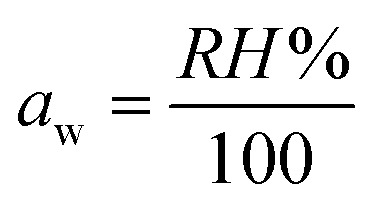


**Fig. 6:**
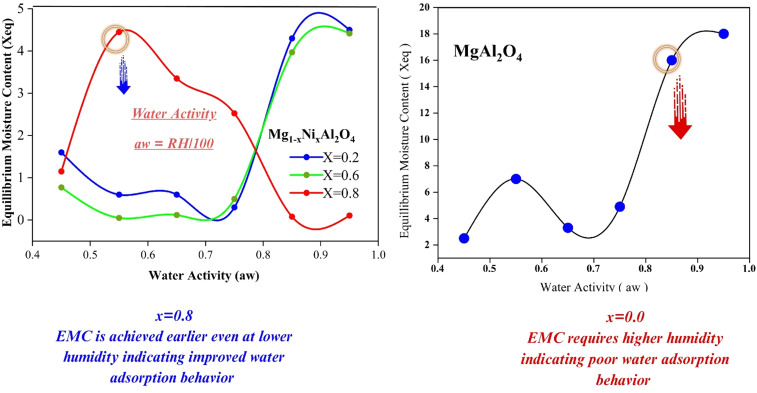
Equilibrium moisture content against water activity (RH/100) for Nickel doped magnesium aluminate spinel having general formula Mg_1−*x*_Ni_*x*_Al_2_O_4_ (*x* = 0.0, 0.2, 0.6, 0.8).

And in the case when *x* = 0.2 almost same behavior as composition when *x* = 0.2, but the only difference is that it has all its equilibrium moisture content values one step higher. The comparison between equilibrium moisture content and water activity of different doped nickel content is discussed in Table 2. By plotting the moisture content trend with respect to time, equilibrium moisture content *X*_eq_ points were obtained at different relative humidity. Whereas water activity “*a*_w_” is the ratio between the vapor pressure on the adsorbent to the vapor pressure of pure water. The graph between water activity (*a*_w_) and equilibrium moisture content is shown in Fig. 6.

**Table 2:** Equilibrium moisture content against water activity (RH/100) for nickel-doped magnesium aluminate spinel having general formula Mg_1−*x*_Ni_*x*_Al_2_O_4_ (*x* = 0.0, 0.2, 0.6, 0.8)

*a*_w_ ± 0.05 (RH/100)	*X*_eq_ (mg g^−1^) ± 0.1 *x* = 0.0	*X*_eq_ (mg g^−1^) ± 0.1 *x* = 0.2	*X*_eq_ (mg g^−1^) ± 0.1 *x* = 0.6	*X*_eq_ (mg g^−1^) ± 0.1 *x* = 0.8
0.45	2.5	1.6	2.7	8.0
0.55	7.0	0.6	0.6	20.0
0.65	4.9	0.7	0.8	16.0
0.75	16.0	0.3	1.9	13.0
0.85	18.0	4.3	12.0	4.1
0.95	18.2	4.5	13.3	4.2

### 3.3 Adsorption isotherm models

**Fig. 7:**
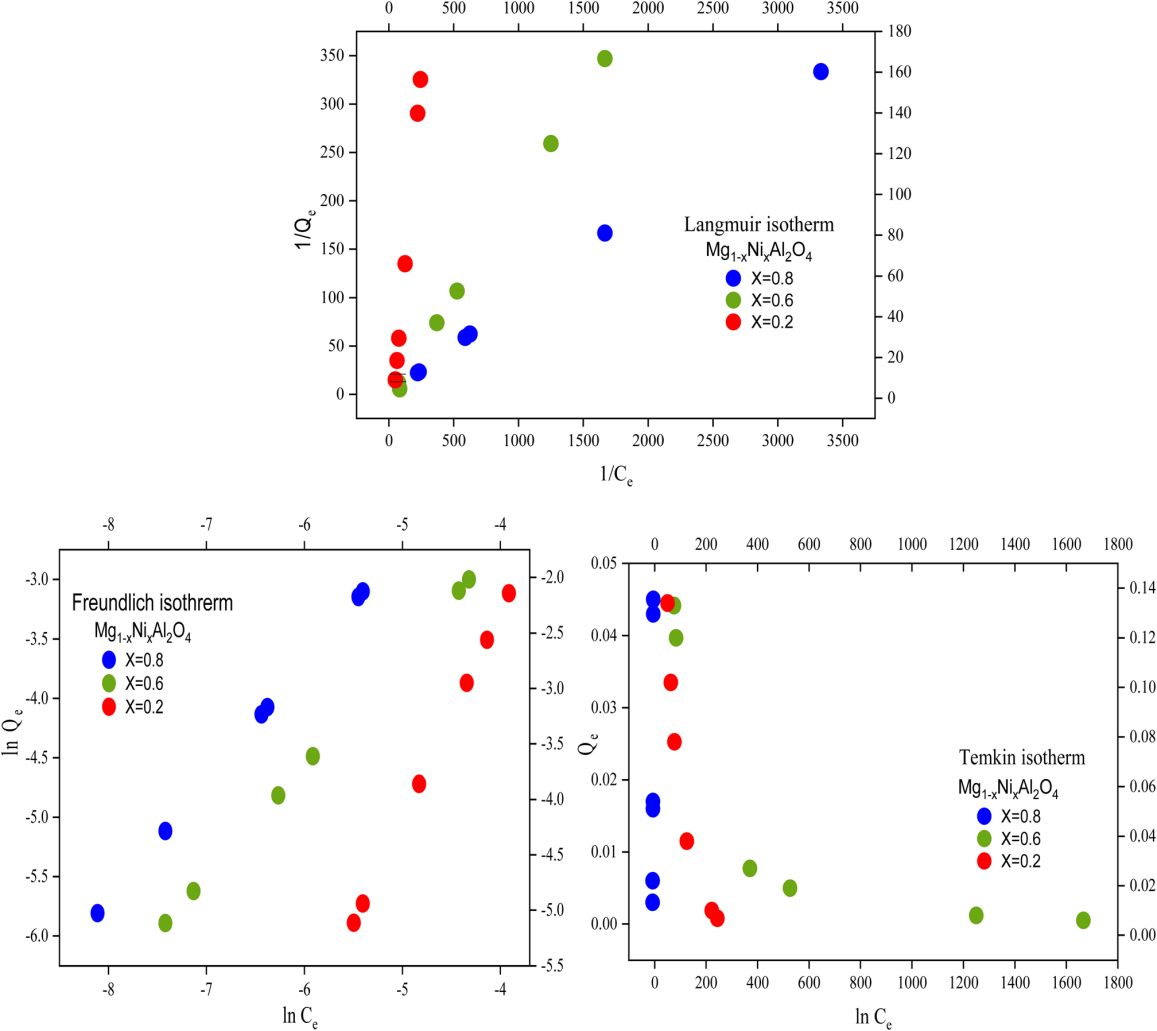
Adsorption behavior of Mg_1−*x*_Ni_*x*_Al_2_O_4_ (*x* = 0.2, 0.4, 0.6) fitted using various isotherm models.

Adsorption isotherm model has significant importance on its own. Such as Langmuir adsorption isotherm about linear adsorption, while Freundlich and Temkin gives information about physisorption or chemisorption (Fig. 7).

#### 3.3.1 Langmuir isotherm model

A key parameter of the Langmuir adsorption isotherm is the separation factor, or dimensionless constant *R*_L_, which is expressed by the following equation.16
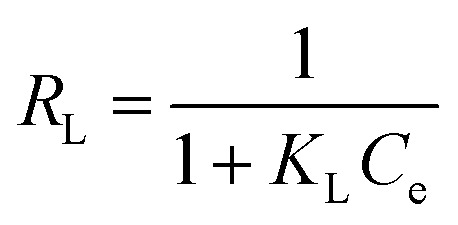


This separation factor has following value assumption. If *R*_L_ > 1, it indicates unfavorable adsorption in which the desorption rate is greater than the adsorption rate. If *R*_L_ = 1. It indicates the process of linear adsorption in which dependency of concentration of adsorbate that adsorb. If *R*_L_ = 0, it indicates strong adsorption means the process is irreversible. Mostly 0 < *R*_L_ < 1, indicates the normal favorable adsorption.^22^

#### 3.3.2 Freundlich model

Freundlich adsorption isotherm is represented by the following equation.17
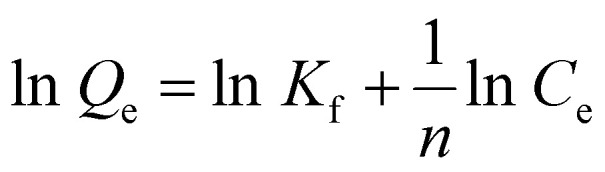
where “*K*_f_” is constant of Freundlich adsorption isotherm, *C*_e_ is the equilibrium point concentration of adsorbate (mg L^−1^), “*Q*_e_” is the concentration of adsorbate that is adsorb on adsorbent (mg g^−1^), and *n* describes the linearity of adsorption process between adsorbate and adsorbent. If *n* = 0, it indicates the linear adsorption process. If *n* > 1, it indicates the chemical adsorption. If *n* < 1, it indicates the physical adsorption^22^

Temkin adsorption isotherm assumes that the heat of adsorption decreases linearly with the surface coverage of adsorbate on adsorbent. There are uniform binding energies on the adsorbent bed during the adsorbate–adsorbent interactions (Table 3).

**Table 3:** Isothermal modeling parameters for Mg_1−*x*_ Ni_*x*_Al_2_O_4_ (*x* = 0.0, 0.2, 0.6, 0.8)

Mg_1−*x*_ Ni_*x*_Al_2_O_4_	Langmuir		Freundlich		Temkin	
Parameters	*K*_L_	*R*_L_	*n*	1/*n*	*B*_T_ (kJ mol^−1^)	*A*_T_
*x* = 0.0	2.3 ± 0.2	0.8 ± 0.2	1 ± 0.5	1 ± 0.5	—	—
*x* = 0.2	2.3 ± 0.2	0.9 ± 0.2	1 ± 0.6	1 ± 0.6	−6.88 ± 0.4	0.9 ± 0.4
*x* = 0.6	2.3 ± 0.3	0.8 ± 0.3	1 ± 0.5	1 ± 0.5	−7.18 ± 0.3	12 ± 0.3
*x* = 0.8	2.3 ± 0.2	0.8 ± 0.2	1 ± 0.7	1 ± 0.7	0.08 ± 0.4	1.0 ± 0.4

#### 3.3.3 Temkin model

Temkin adsorption isotherm assumes that the heat of adsorption decreases linearly with the surface coverage of adsorbate on adsorbent. There are uniform binding energies on the adsorbent bed during the adsorbate–adsorbent interactions.

Temkin adsorption isotherm is represented by the following equation.

18 *Q*_e_ = *B*_T_*L*_*n*_*A*_T_ + *B*_T_ ln *C*_e_

where “*B*_T_” is the heat constant of adsorption (if the *B*_T_ < 8 kJ mol^−1^, it indicates the physical adsorption “*A*_T_” is the equilibrium binding constant of adsorption, and *T* is the absolute temperature.^22^

### 3.4 Kinetic studies

A plot between log *M*_*t*_ (mg g^−1^) *versus* the time (min) gives straight line with minimum error ±3 gives intercept values, *M*_0_, *M*_e_, and first order rate constants. The linear regression correlation coefficient *R*^2^ obtained good value at first order kinetics in comparison with second order kinetics. They obtained good comparison between the theoretical and experimental parameters especially in higher content doped nickel in magnesium aluminate spinel *x* = 0.8. The first order kinetics tells the synthesized adsorbent showed physical adsorption, result that the process can be reverse as desorption without high energy demands, thus solar-driven. The first order followed more linearly at *x* = 0.8 with relative humidity values of 45% and 55%, and linearity decreases with higher humidity due to reason of decrease diffusion rate of moisture towards the surface of the material.

**Fig. 8:**
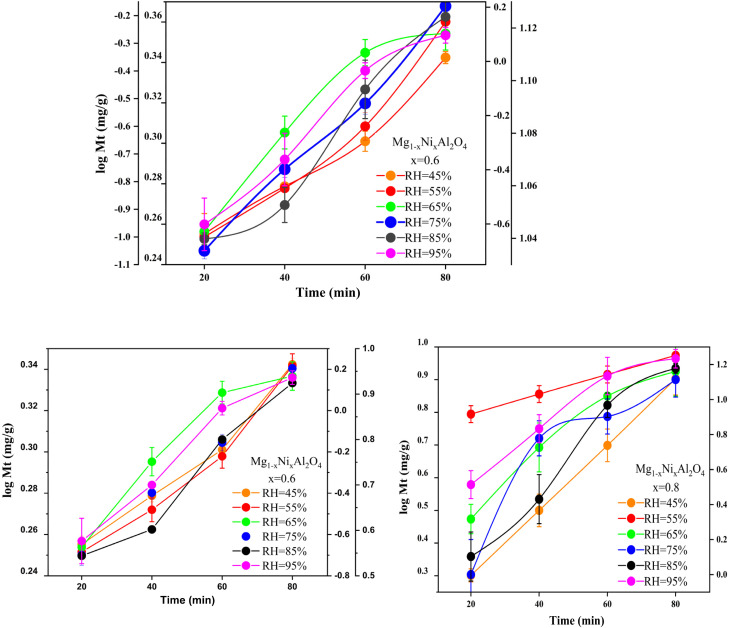
First-order kinetic modeling of Mg_1−*x*_Ni_*x*_Al_2_O_4_ (*x* = 0.2, 0.4, 0.6) adsorption at different relative humidities.

The value of *k*_1_ decreases by increasing humidity as in lower humidity the equilibrium established smoothly with linear trend and moisture occupied the adsorption sites by physical adsorption. While at higher humidity, the equilibrium established fast in short time duration, as all sites occupied earlier and no further adsorption sites available at adsorbent surfaces. The plotting of first order kinetics of various nickel doped content is following in Fig. 8.

### 3.5 Thermodynamic study

#### 3.5.1 Adsorption isosteric heat

The isosteric heat of adsorption (*Q*_st_) refers to the energy liberated during sorption. It is defined as the combined value of the net isosteric heat of adsorption (*q*_st_) and the heat of vaporization of water, HL (40.65 kJ mol^−1^).^12^

19 *Q*_st_ = *q*_st_ + *H*_L_

Experimentally, the isosteric heat of adsorption can be obtained from the Clausius–Clapeyron relationship by constructing a plot of ln(water activity) *versus* 1/*T*.20
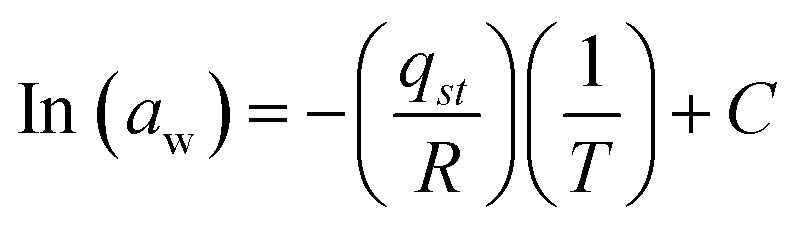
Here, *T* is the absolute temperature (K), *R* is the gas constant (8.314 J mol^−1^ K^−1^) “*q*_st_” is the net isosteric heat of sorption (J mol^−1^) and *C* is the constant.

The isosteric heat value of *Q*_st_ = −21.805 kJ mol^−1^ obtained by the use of eqn (20) is calculated from the slope.

#### 3.5.2 Thermodynamic entropy of sorption

The change in entropy provides insights into the energy aspects of the system and is directly related to the number of adsorption sites available. The differential entropy of sorption (Δ*S*) was obtained by applying eqn (14) to the equilibrium moisture content data derived from the fitting equation.^12^21
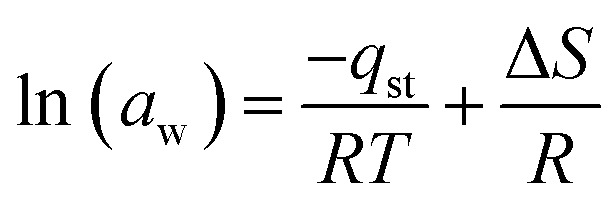
*c* = −(27.20); *R* = (8.314) J mol^−1^ k^−1^22
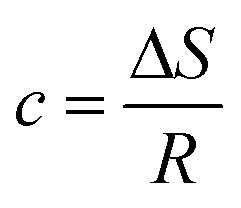
Δ*G =* −0.2261 KJ mol^−1^

#### 3.5.3 Thermodynamic Gibbs free energy

Gibbs free energy quantifies the energy change during adsorption at constant pressure and temperature, indicating whether the process occurs spontaneously or not. The Gibbs free energy “Δ*G*”, calculated at temperature of 16.4 °C and 75% relative humidity by following equation.

23 Δ*G* = *R*_T_ ln *a*_w_

Δ*G =* −0.2261 KJ mol^−1^

As the Gibbs free energy Δ*G* value is negative, indicated adsorption process is spontaneous.

**Fig. 9:**
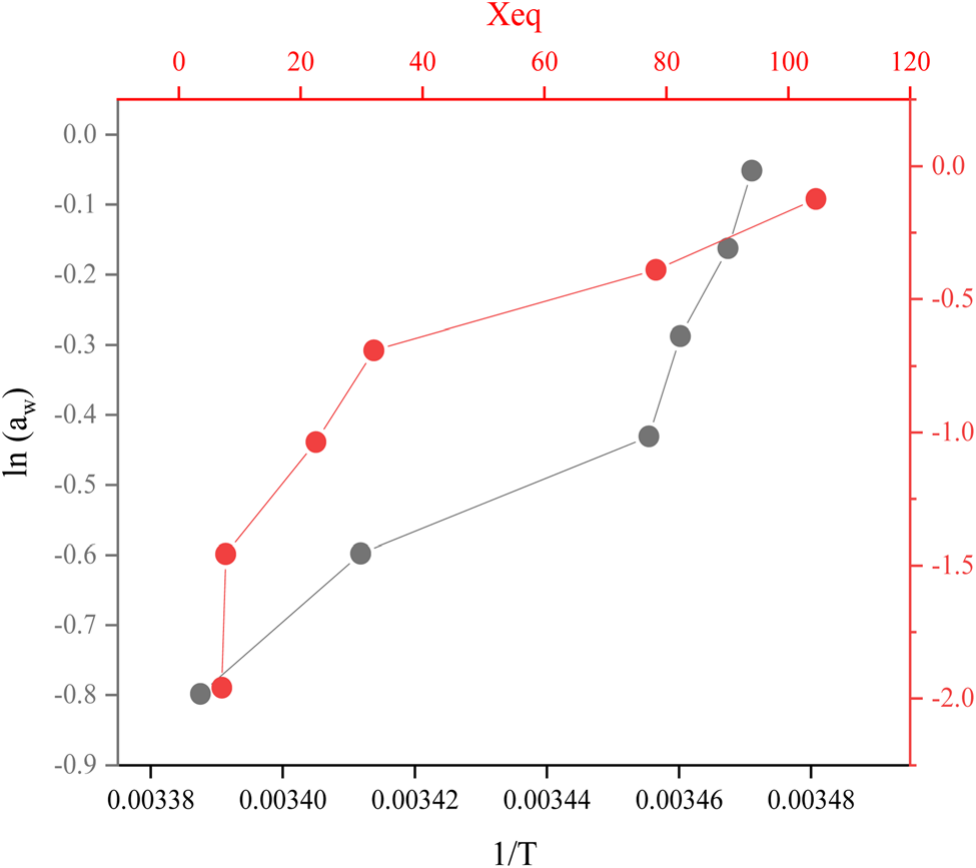
Plot between ln *a*_w_*versus* 1/*T* and change in Gibbs free energy with respect to *X*_eq_.

It is observed the Δ*G* value with the increase in the equilibrium moisture content increases. Δ*G* varied between −1.95 and −0.12 kJ mol^−1^, and the negative values confirm that adsorption occurs spontaneously under all tested relative humidity conditions (Fig. 9).

## 4. Conclusion

SBAWH was carried out by Ni-doped MAS sorbent. MAS and doped derivatives were successfully synthesized by the co-precipitation method. Synthesized material shows a single-phase spinel confirmed by XRD analysis while morphology and elemental analysis was confirmed by SEM. The successful incorporation of Ni into MAS was evaluated by both XRD and EDX analysis. Surface charges in spinel play an important role in water harvesting capabilities of doped spinel. The increased amount of dopant increases the defects in the structure which leads to an increase in water adsorption characteristics of synthesized materials. The adsorption behavior of the material was carried out by using a small setup by varying humidity of 45–95%. The relative humidity shows a direct relation with water adsorption capacity. When the relative humidity increased from 45% to 85%, the water adsorption capacity of Mg_1−*x*_Ni_*x*_Al_2_O_4_ changed noticeably. For the sample with *x* = 0.2, the capacity rose from 1.6 mg g^−1^ to 2.7 mg g^−1^, whereas for *x* = 0.6, it increased from 4.3 mg g^−1^ to 12 mg g^−1^ respectively. And *x* = 0.8 has shown EMC at lower humidity value as 20 mg g^−1^ while *x* = 0.0 has shown EMC very late at higher humidity value as 18 mg g^−1^. The sample with *x* = 0.8 composition showed water adsorption capacity of 8 mg g^−1^ to 13 mg g^−1^ at 45–75% RH. Kinetic and thermodynamic studies indicate that the adsorption process is exothermic and exhibits physisorption behavior. While this study highlights the fascinating water adsorption potential of Ni-doped magnesium aluminate spinel at the lab level, it does face some limitations. Specifically, there's a need for more detailed textural characterization, long-term cycling data, and performance assessments in varying environmental conditions. Moving forward, the research will not only focus on testing the material in real-world atmospheric water harvesting systems but will also dive deeper into thorough BET and pore characterization, optimizing the dopant levels, and examining the adsorption–desorption behavior. These efforts will provide crucial insights into the material's stability, regeneration efficiency, and overall performance for large-scale water harvesting applications.

## Author contributions

Afsah Saleem: conceived and designed the experiments, performed the experiments, analyzed the data, prepared figures and/or tables, write the original draft. Raouf Hassan: reviewing, validation, data acquisition, and funding. Muhammad Ehtisham: conceived and designed the experiments, validation, and data acquisition. Mansour S. Almatawa: data acquisition, and funding. Ahmad K. Badawi: authored and reviewed drafts of the article, sources, validation, analysis, editing and reviewing and approved the final draft. Bushra Ismail*: supervision, provided materials, conceived and designed the experiments, analyzed the data, authored or reviewed drafts of the article, validation, and approved the final draft.

## Conflicts of interest

There are no conflicts to declare

## Funding

This work was supported and funded by the Deanship of Scientific Research at Imam Mohammad Ibn Saud Islamic University (IMSIU) (grant number IMSIU-DDRSP2602).

## Supplementary Material

RA-016-D5RA09380E-s001

## Data Availability

All data supporting this article have been provided within the main content of this manuscript. No additional data are available outside of this publication. Supplementary information (SI): the synthesis methodology and the designed compositions of Ni^2+^ doped samples of MAS. See DOI: https://doi.org/10.1039/d5ra09380e.
