# Supplementary figures and images for: Influence of nickel doping on the moisture adsorption properties of magnesium aluminate spinel: thermodynamic and kinetic analysis
Source: RSC Adv. 2026 Feb 17;16(11):9399–411. doi: 10.1039/d5ra09380e (PMC12910558; doi:10.1039/d5ra09380e)

## Supplementary data

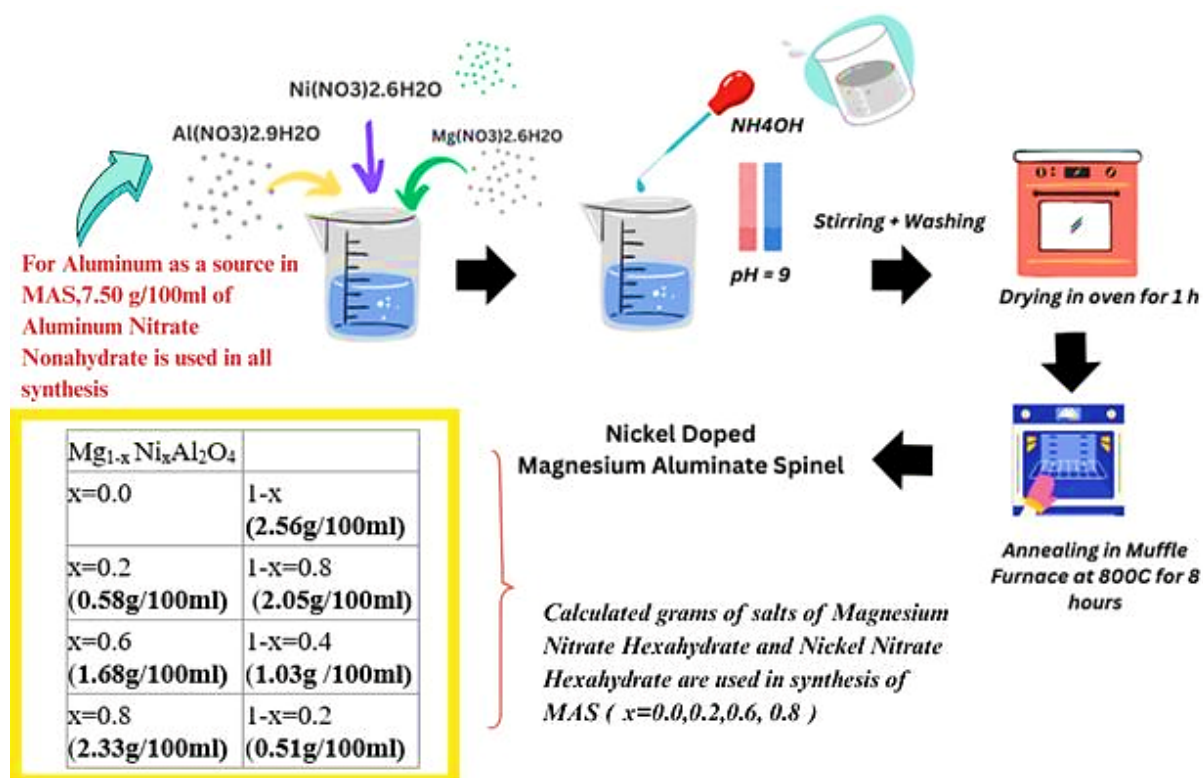

**Figure S1:** Scheme for synthesis of  $\text{Mg}_{1-x}\text{Ni}_x\text{Al}_2\text{O}_4$  ( $x=0.0, 0.2, 0.6, 0.8$ )

Supplement: RA-016-D5RA09380E-s001 [file RA-016-D5RA09380E-s001.pdf]
